# Effect of Nonlinear Elasticity on the Swelling Behaviors of Highly Swollen Polyelectrolyte Gels

**DOI:** 10.3390/gels7010025

**Published:** 2021-03-01

**Authors:** Jian Tang, Takuya Katashima, Xiang Li, Yoshiro Mitsukami, Yuki Yokoyama, Ung-il Chung, Mitsuhiro Shibayama, Takamasa Sakai

**Affiliations:** 1 Department of Bioengineering, Graduate School of Engineering, The University of Tokyo, Tokyo 113-8656, Japan; tang@tetrapod.t.u-tokyo.ac.jp (J.T.); tei@tetrapod.t.u-tokyo.ac.jp (U.-i.C.); 2 The Institute for Solid State Physics, The University of Tokyo, Chiba 227-8581, Japan; x.li@issp.u-tokyo.ac.jp; 3 Superabsorbents Research Department, Nippon Shokubai Co. Ltd., Hyogo 671-1292, Japan; yoshiro_mitsukami@shokubai.co.jp (Y.M.); yuki_yokoyama@shokubai.co.jp (Y.Y.); 4 Neutron Science and Technology Center, Comprehensive Research Organization for Science and Society, Ibaraki 319-1106, Japan; m_shibayama@cross.or.jp

**Keywords:** polyelectrolyte, polymer gel, swell, Gibbs–Donnan effect, counterion condensation, nonlinear elasticity, Neo–Hookean model, Gent model

## Abstract

Polyelectrolyte gels exhibit swelling behaviors that are dependent on the external environment. The swelling behaviors of highly charged polyelectrolyte gels can be well explained using the Flory–Rehner model combined with the Gibbs–Donnan effect and Manning’s counterion condensation effect (the FRGDM model). This study investigated the swelling properties of a series of model polyelectrolyte gels, namely tetra-polyacrylic acid-polyethylene glycol gels (Tetra-PAA-PEG gels), and determined the applicability of the FRGDM model. The swelling ratio (*V*_s_/*V*_0_) was well reproduced by the FRGDM model in the moderate swelling regime (*V*_s_/*V*_0_ < 10). However, in the high swelling regime (*V*_s_/*V*_0_ > 10), the FRGDM model is approx. 1.6 times larger than the experimental results. When we introduced the finite extensibility to the elastic free energy in the FRGDM model, the swelling behavior was successfully reproduced even in the high swelling regime. Our results reveal that finite extensibility is one of the factors determining the swelling equilibrium of highly charged polyelectrolyte gels. The modified FRGDM model reproduces well the swelling behavior of a wide range of polyelectrolyte gels.

## 1. Introduction

Polyelectrolyte gels contain fixed ions on their polymer chains and mobile ions within the solvent. Generally, polyelectrolyte gels exhibit a significant swelling ability, wherein the swelling ratio depends on the external solution, and under the appropriate conditions, can be sufficiently large [1,2,3,4]. In polymer gels, the polymer chains are microscopically solvated, generating a mixing free energy. However, since the polymer gel is solid, it exhibits a restoring force against deformation. As a result, the balance between the mixing free energy ($F_{\mathrm{mix}}$) and the elastic free energy ($F_{\mathrm{el}}$) results in the equilibrium swelling state. In polyelectrolyte gels, the fixed ions present on the network strands cannot diffuse, generating an imbalanced distribution of mobile ions inside and outside the gel; this is known as the Gibbs–Donnan effect. Such an imbalanced distribution of mobile ions induces an additional free energy related to swelling of the gel ($F_{\mathrm{ion}}$). $F_{\mathrm{ion}}$ is influenced by the p*K*a of the fixed ions, thereby resulting in polyelectrolyte gels that exhibit a pH-responsive swelling behavior. Due to the unique swelling properties of polyelectrolyte gels, many studies have been conducted to understand the relationship between the distribution of fixed ions and the swelling properties.

Previously, Rička and Tanaka [5] established a pioneering model for describing the swelling behaviors of polyelectrolyte gels by combing the Flory–Rehner model with the Gibbs–Donnan effect (i.e., the FRGD model). According to the Flory–Rehner model, the total free energy comprises the elastic and mixing free energies. The elastic free energy is expressed by the Neo–Hookean function [6,7], where a regular network composed of Gaussian chains with uniform length is assumed. On the other hand, the mixing free energy is estimated using the lattice approximation [8,9]. Tanaka et al. calculated the ionic free energy using the Gibbs–Donnan effect by assuming that the electroneutrality is preserved within the gel, and that the activities of the mobile ions are equal inside and outside the gel [10]. The polyelectrolyte gel reaches an equilibrium swelling state when the sum of the three free energies is minimized [6]. This model successfully described the swelling behaviors of weakly charged polyelectrolyte gels [11,12,13,14], but it failed for highly charged polyelectrolyte gels due to overestimation of the ionic pressure contribution [15,16]. This overestimation has often been explained in the framework of the counterion condensation effect. When the charge density exceeds a critical value, a portion of the counterions is trapped around a network strand and mobility is lost, resulting in a reduction in the effective fixed ion concentration [17]. Although a few studies have attempted to combine the FRGD model with the counterion condensation effect, a deviation remained between the experimental results and the predictions [2,3,18,19,20]. Although conventional studies utilized polyelectrolyte gels prepared through the random copolymerization of charged monomers and neutral crosslinkers [21], it was not possible to control the spatial distribution of charged monomers and neutral crosslinkers in these systems, thereby preventing a precise estimation of the counterion condensation effect. The validation of such theoretical models has therefore yet to be achieved.

Previously in our group, we fabricated a polymer gel with a well-controlled network structure based on the combination of two tetra-armed poly (ethylene glycol)s through an AB-type crosslink-coupling reaction, and this was named a Tetra-PEG gel [22,23,24]. As the tetra-armed building blocks were precisely synthesized via anionic polymerization, the resultant networks were expected to have a regular network structure with tetrafunctional cross-linking points and a uniform strand length. Recently, we utilized the same strategy for the preparation of polyelectrolyte gels and successfully designed a regular polyelectrolyte gel (Tetra-PAA-PEG gel) possessing alternating neutral/highly charged sequences by a combination of a tetrathiol-terminated poly (ethylene glycol) (Tetra-PEG-SH) and a tetramaleimide-terminated poly (acrylic acid) (Tetra-PAA-MA) via click chemistry. According to the molecular design, the neutral and charged segments are expected to be alternately connected with one another. The network homogeneity in this gel was examined by spectroscopic and mechanical measurements, and it was found that the Tetra-PAA-PEG gel was a promising model for investigating the physicochemical properties of highly charged polyelectrolyte gels. In our previous study, we also investigated the swelling behaviors of the Tetra-PAA-PEG gel in various tuned external solutions, and validated the model describing the swelling behaviors of the Tetra-PAA-PEG gel through modification of the FRGD model with Manning’s counterion condensation model (i.e., the FRGDM model) [25].

However, in our previous work, validation of the FRGDM model remained limited due to the fact that we tested only a Tetra-PAA-PEG gel with a uniform network structure, a constant crosslink density, and a constant polymer volume fraction in the as-prepared state. In addition, there have been some reports that the nonlinear elastic effect of the network strands becomes significant, especially in the highly swollen state [26,27,28,29].

Thus, to further examine the FRGDM model, we herein employ tuning of the elastic moduli to maintain the crosslinking density. This unconventional control over elastic moduli is enabled by the ‘negative energy elasticity,’ which softens hydrogels, and was recently discovered by our group [30]. More specifically, we tune the molecular weights of neutral Tetra-PEG units to maintain a constant crosslinking density, and prepare a series of gels with tuned elastic moduli. In addition, we investigate the swelling behaviors of these gels and compare them with the FRGDM model to determine the applicability of this model to reproduce the swelling behaviors of polyelectrolyte gels. Based on our experimental study, we show the limitation of the FRGDM model, and propose a modification on the model considering the finite extensibility effect by adopting the Gent model.

## 2. Theoretical Description of the Equilibrium Swelling State

This section introduces several theories that describe the swelling properties of polyelectrolyte gels under different conditions. According to Flory’s assumption [6], the swelling ratio (*Q*) of a neutral hydrogel is divided into two contributions to the swelling pressure of the system: the elastic free energy accompanied by the swelling of the network strands, $\Pi_{\mathrm{el}}$, and the free energy derived from the mixing of polymer segments with solvent molecules, $\Pi_{\mathrm{mix}}$. In polyelectrolyte gels, the existence of ionic groups fixed on the polymer chains leads to an imbalanced distribution of the counterions inside and outside the gel, which is known as the Gibbs–Donnan effect. The Gibbs–Donnan effect contributes to the swelling pressure $\Pi_{\mathrm{ion}}$ [31,32]. Thus, to obtain the equilibrium swelling state, the total pressure of the polyelectrolyte gels is defined as [33]:

(1) $$\Pi =\Pi_{\mathrm{el}}+\Pi_{\mathrm{mix}}+\Pi_{\mathrm{ion}}=0$$

### 2.1. Estimation of the Elastic Pressure $\Pi_{el}$

The Neo–Hookean model describes the elastic free energy under appropriate deformation [7,34,35], and the elastic pressure is expressed as:

(2) $$\Pi_{\mathrm{el}}=-G_{0}Q^{-\frac{1}{3}}$$

where $G_{0}$ is the shear modulus in the as-prepared state, and $G_{0}Q^{-1/3}$ is equal to the shear modulus in the swollen state.

### 2.2. Estimation of the Mixing Pressure $\Pi_{mix}$

Assuming the polymerization degree of a gel to be infinite, $\Pi_{\mathrm{mix}}$ is obtained by applying the lattice approximation based on the Flory-Huggins theory and can be expressed as [6,8,9]:

(3) $$\Pi_{\mathrm{mix}}=-\frac{RT}{V_{1}}\left[\ln(1-\phi )+\phi +\chi \phi^{2}\right]=-\frac{RT}{V_{1}}\left[\ln\left\{1-\left(\frac{\phi_{0}}{Q}\right)\right\}+\left(\frac{\phi_{0}}{Q}\right)+\chi {\left(\frac{\phi_{0}}{Q}\right)}^{2}\right]$$

where *R* is the gas constant, *T* is the absolute temperature, $V_{1}$ is the molar volume of the solvent, $\phi_{0}$ is the polymer volume fraction in the as-prepared state, ϕ is the polymer volume fraction in the swollen state ($Q=\phi_{0}/\phi$), and χ is the Flory-Huggins interaction parameter.

### 2.3. Estimation of the Ionic Pressure $\Pi_{ion}$

Polyelectrolyte gels possess fixed ions on their polymer chains. Mobile counterions diffuse and balance the chemical potentials between the inside and outside of the gel, and a portion of the counterions inside the gel neutralizes the fixed ions on the polymer chain. As a result, the distributions of the mobile ion concentrations inside and outside the gel are imbalanced. Thus, $\Pi_{\mathrm{ion}}$ is given by [31]:

(4) $$\Pi_{\mathrm{ion}}=RT\underset{i}{\sum }\left(C_{i}-C_{i}{}^{\prime }\right)$$

where $C_{i}$ and $C_{i}{}^{\prime }$ are the concentrations of the mobile ions inside and outside of the gel, respectively. Here, subscript *i* indicates the type of mobile ion. Donnan [36] described the relationship between the concentration ratio of the mobile ions inside and outside the gel, which is given by:

(5) $$\frac{C_{i}}{C_{i}{}^{\prime }}=K^{Z_{i}}$$

where *K* is the so-called Gibbs–Donnan ratio, and $Z_{i}$ is the valency of the mobile ions. It is worth noting that the Gibbs–Donnan ratio *K* is larger than 1 when the fixed ions on the polymer chain are negatively charged. This indicates that the ionic pressure contributes to the swelling of polyelectrolyte gels [5]. The total concentration of the mobile ions inside the gel ($C_{m}$) was estimated based on Equation (5) and is given by:

(6) $$C_{m}=\underset{i}{\sum }Z_{i}K^{Z_{i}}C_{i}{}^{\prime }$$

The fixed ion concentration inside the gel ($C_{f}$) is defined by the dissociation equilibrium constant (*K*_a_). In this study, the fixed group is the carboxyl group (-COOH), and so the concentration of ionized carboxyl groups can be expressed as:

(7) $$\left[{\mathrm{COO}}^{-}\right]=\frac{{[\mathrm{COOH}]}_{0}}{10^{pK_{a}-\mathrm{pH}}+1}$$

where ${[\mathrm{COOH}]}_{0}$ is the total concentration of fixed ions. The concentration of fixed ions inside the gel $C_{f}$ is given by:

(8) $$C_{f}\;=\;Z_{a}\frac{{[\mathrm{COOH}]}_{0}}{Q(1+\frac{KC_{H}{}^{\prime }}{K_{a}})}$$

where *Z*_a_ is the valence of the fixed ions and $C_{H}{}^{\prime }$ is the concentration of protons in the external solution. The environment inside the gel should be electroneutral. Thus, the sum of Equations (6) and (8) should be equal to 0, which is expressed as:

(9) $$\underset{i}{\sum }Z_{i}K^{Z_{i}}C_{i}{}^{\prime }+Z_{a}\frac{{[\mathrm{COOH}]}_{0}}{Q(1+\frac{KC_{H}{}^{\prime }}{K_{a}})}=0$$

In this Equation (9), the first term on the left side is the concentration of mobile ions, and the second term is the concentration of fixed ions on the polymer chain.

### 2.4. Estimation of the Swelling Ratio Q by the Flory–Rehner Model Considering the Gibbs–Donnan Effect and Manning’s Counterion Condensation Effect (the FRGDM Model)

The FRGD model can describe the swelling behaviors of weakly charged polyelectrolyte gels when the average contour distance between the neighboring fixed charges of a polymer chain (*b*) is larger than the Bjerrum length (*l*_B_) [17]. *l*_B_ is the separation when the electrostatic energy between the unit electrostatic charges becomes equal to the thermal energy. When the solvent and ion species are fixed to water and sodium, *l*_B_ is 7.14 Å at 298 K [37].

On the other hand, in a polyelectrolyte gel with a relatively high charge density (e.g., Tetra-PAA-PEG gel), the distance between neighboring fixed ions is shorter than the Bjerrum length. Here, the binding force between the fixed ions and the counterions becomes more significant than the thermal fluctuation. Thus, the counterions are localized around the network strands, resulting in a decrease in the effective fixed ion concentration [COOH]_0,effective_ [38,39], which represents counterion condensation. Manning [17,40] described the counterion condensation in a monovalent salt solution with modification of the effective fixed ion concentration, which is given by:

(10) $${\left[\mathrm{COOH}\right]}_{0,\;\mathrm{effective}}=\frac{{\left[\mathrm{COOH}\right]}_{0}}{2\xi }$$

where *ξ* is a characteristic size defined by Manning, and is given by $\xi =l_{B}/b$. Using the modification of Equation (10), Equation (9) can be rewritten as

(11) $$\underset{i}{\sum }Z_{i}K^{Z_{i}}C_{i}{}^{\prime }+Z_{a}\frac{{[\mathrm{COOH}]}_{0}}{2Q\xi (1+\frac{KC_{H}{}^{\prime }}{K_{a}})}=0$$

Using Equation (11), *K* can be described as a function of Q depending on the pH. The relationship between *K* and Q is obtained by substituting Equations (2)–(4) into Equation (1) as follows:

(12) $$-G_{0}Q^{-\frac{1}{3}}-\frac{RT}{V_{1}}\left[\ln\left\{1-\left(\frac{\phi_{0}}{Q}\right)\right\}+\left(\frac{\phi_{0}}{Q}\right)+\chi {\left(\frac{\phi_{0}}{Q}\right)}^{2}\right]+\;RT\underset{i}{\sum }C_{i}^{\prime }(K^{Z_{i}}-1)=\;0$$

where *K* and Q are determined to satisfy Equations (11) and (12).

## 3. Results and Discussion

### 3.1. Fabrication of Tetra-PAA-PEG Gels with Tuned Neutral Segment Lengths

**Figure 1:**
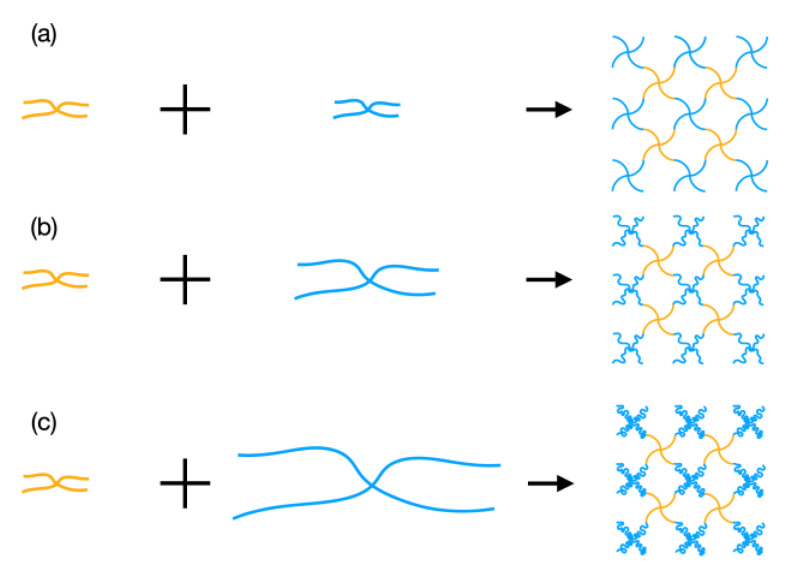
Schematic illustration of the design of the tetra-poly (acrylic acid)-poly (ethylene glycol) gels (Tetra-PAA-PEG gels) with tuned neutral segment lengths, where the orange and blue parts represent the poly (acrylic acid) and poly (ethylene glycol) units, respectively. (**a**) 10 K Tetra-PAA-PEG gel; (**b**) 20 K Tetra-PAA-PEG gel; and (**c**) 40 K Tetra-PAA-PEG gel.

To obtain a series of elastic moduli in the as-prepared state, we fabricated Tetra-PAA-PEG gels with tuned neutral segment lengths, as shown in Figure 1. Here, we fixed the initial crosslink density and fixed ion concentration by using an identical molar concentration and molecular weight of the Tetra-PAA polymer. In citric-phosphatase buffer, Tetra-PAA with a *M*_w_ = 19 kg mol^−1^ and Tetra-PEG with *M*_w_ values of 10, 20, and 40 kg mol^−1^ were mixed. The molar concentrations of the total prepolymers and the monomeric PAA units of the Tetra-PAA-PEG gels in the as-prepared state were set to be 3.2 × 10^−3^ and 4.2 × 10^−1^ mol L^−1^, respectively. To control the gelation time, the pH and ionic strength of the buffer were set to 5.9 and 100 mM, respectively.

**Figure 2:**
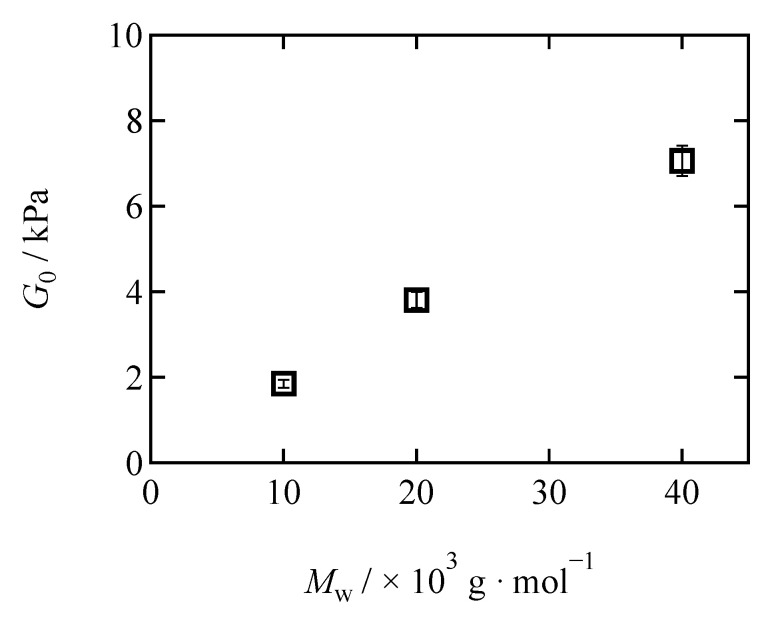
The molecular weight (*M*_w_) dependence of the shear modulus ($G_{0}$) for the Tetra-PAA-PEG gels.

Figure 2 shows the molecular weight dependence of the shear moduli of the Tetra-PAA-PEG gels. As indicated, *G*_0_ increased with increasing *M*_w._ In general, the elasticity of gels is composed of two contributions, namely the entropic and energetic contributions. According to our recent study, the energetic contribution of the gel elasticity is negative and can be equivalent to 50% of the entropic contribution [30]. The entropic contribution is determined by the crosslink density [6,41,42,43], while the energetic contribution is determined by the polymer concentration normalized by the overlapping concentration of the prepolymers (overlap factor). In our system, the molar concentration of the crosslinks was set to be identical, and the total polymer concentration increased with increasing network strand length. Thus, higher *M*_w_ specimens exhibit higher overlap factors, resulting in a lower negative energy elasticity and a higher *G*_0_. Notably, the obtained shear moduli agreed well with those of the Tetra-PEG gels at the same overlap factors (see Supporting Information Sections 1 and 2).

### 3.2. Effect of the Neutral Segment Length on the Swelling Behavior

**Figure 3:**
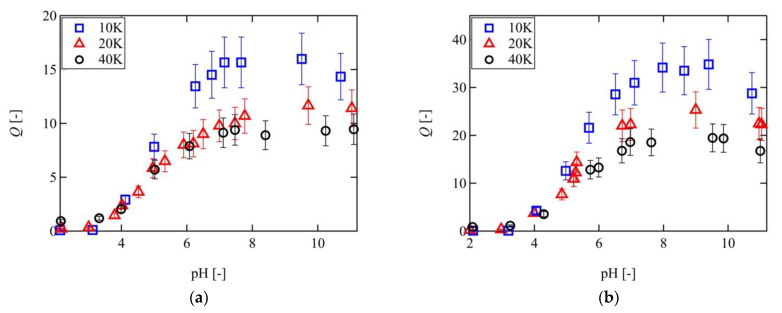
pH dependence of the swelling ratios of the 10 K (blue), 20 K (red), and 40 K (black) Tetra-PAA-PEG gels in salt solutions with different ionic strengths. (**a**) 10 mM, (**b**) 2 mM.

Figure 3 shows the pH dependence of the swelling ratios of the 10 K, 20 K, and 40 K Tetra-PAA-PEG gels in NaCl solutions with ionic strengths of 2 and 10 mM. As indicated, the swelling ratios of all Tetra-PAA-PEG gels increased with increasing pH values, and a plateau was reached when the pH reached > 7.0; this value corresponds to the pH dependence of the ionization of the acrylic acid units present on the polymer chains. In addition, the swelling ratio was found to decrease with increasing ionic strength. The mobile osmotically active ions contribute to the driving force of the swelling process. When the ionic strength increases, more mobile ions diffuse from the external solution into the gel, and the difference between the concentrations of the mobile ions inside and outside the gel decrease, resulting in the decrease of the swelling ratio. These pH and ionic strength dependences of the swelling ratio, therefore, indicate the presence of ionic pressure, as determined from the Gibbs–Donnan effect. It is worth noting that in the case where the Gibbs–Donnan effect works effectively, the salt concentration is larger than the fixed ion concentration.

It was also observed that the swelling ratio of the 10 K Tetra-PAA-PEG gel was almost double those of the 20 K and 40 K Tetra-PAA-PEG gels at pH >7. These swelling behaviors were in contrast to those of the electrically neutral Tetra-PEG gels, where the swelling ratio of the 20 K Tetra-PEG gel was larger than that of the 10 K Tetra- PEG gel under the same $\phi_{0}$ [44]. This contradiction can be explained by the balance between $\Pi_{\mathrm{el}}$ and $\Pi_{\mathrm{mix}}$ for the Tetra-PAA-PEG gel (see Section 3.3).

### 3.3. Effect of the Neutral Segment Length on the Mechanical Properties

**Figure 4:**
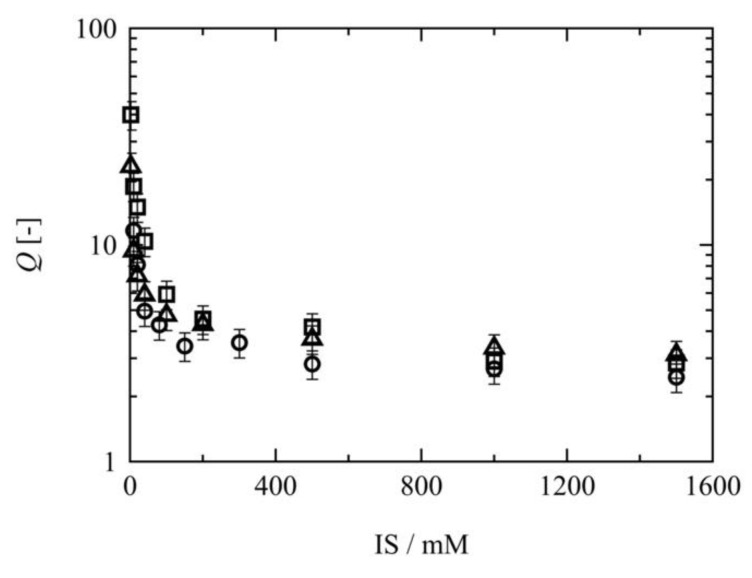
Ionic strength dependences of the swelling ratios of the Tetra-PAA-PEG gels in NaCl solutions: 10 K Tetra-PAA-PEG gel (squares), 20 K Tetra-PAA-PEG gel (circles), and 40 K Tetra-PAA-PEG gel (triangles). The pH values of the outer solutions for the 10 K, 20 K, and 40 K Tetra-PAA-PEG gels were 8.6, 9.7, and 8.0, respectively.

As mentioned in the theoretical section, the balance among $\Pi_{\mathrm{el}}$, $\Pi_{\mathrm{mix}}$, and $\Pi_{\mathrm{ion}}$ determines the swelling ratio of a polyelectrolyte gel. Here, we set the same fixed ion concentration among the 10 K, 20 K, and 40 K Tetra-PAA-PEG gels. It was found that $\Pi_{\mathrm{ion}}$ was consistent for all Tetra-PAA-PEG gels under equal values of *Q*, and so the sum of $\Pi_{\mathrm{el}}$ and $\Pi_{\mathrm{mix}}$ determines the order of the swelling ratios. In addition, $\Pi_{\mathrm{el}}$ is determined using the shear modulus in the as-prepared state in combination with the swelling ratio, while $\Pi_{\mathrm{mix}}$ is determined by the *χ* parameter, the initial polymer concentration, and the swelling ratio. The *χ* parameter was experimentally estimated from *Q* at pH > 8.0 and using a 1500 mM NaCl solution, where the effects of hydrogen bonding between the PAA and PEG chains [45,46,47] and the electrostatic potential from the fixed ions are negligible. It was found that the swelling ratios were relatively constant under the high ionic strength conditions shown in Figure 4. Under these conditions, the swelling pressure is composed only of the elastic and mixing pressures. Thus, Equation (1) can be rewritten as:

(13) $$\Pi =\Pi_{\mathrm{el}}+\Pi_{\mathrm{mix}}=0$$

Substituting Equations (2) and (3) into Equation (13), the χ parameter was obtained and expressed as:

(14) $$\chi =-\frac{\frac{G_{0}V_{1}Q^{-\frac{1}{3}}}{RT}+\ln\left\{1-\left(\frac{\phi_{0}}{Q}\right)\right\}+\left(\frac{\phi_{0}}{Q}\right)}{{\left(\frac{\phi_{0}}{Q}\right)}^{2}}$$

The χ parameters were therefore calculated to be 0.46, 0.46, and 0.45 for the 10 K, 20 K, and 40 K Tetra-PAA-PEG gels, respectively. It is worth noting that the addition of NaCl had no significant effect on the χ parameter [48].

**Figure 5:**
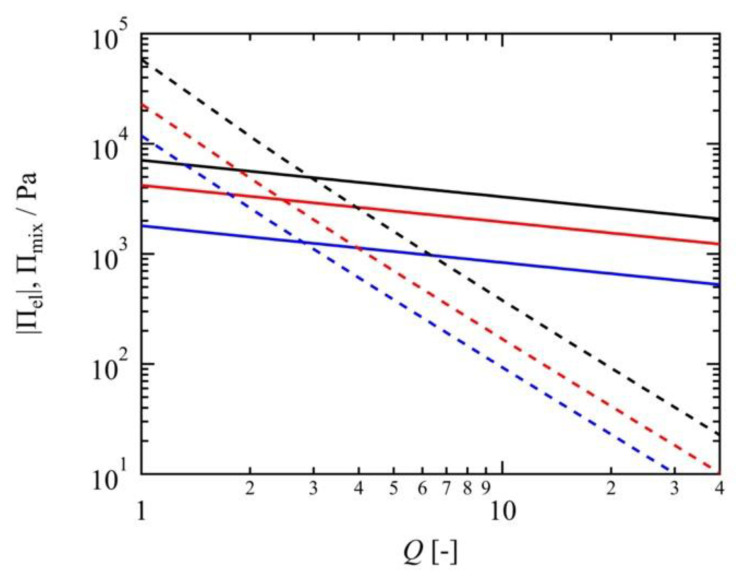
The absolute value of elastic pressure |$\Pi_{\mathrm{el}}$| (solid lines) and mixing pressure $\Pi_{\mathrm{mix}}$ (dashed lines) of the 10 K (blue), 20 K (red), and 40 K (black) Tetra-PAA-PEG gels as a function of the swelling ratio *Q*.

Using the values of the shear moduli and the estimated χ parameters, we can simulate $\Pi_{\mathrm{el}}$ and $\Pi_{\mathrm{mix}}$ as a function of *Q*, as shown in Figure 5. When *Q* is larger than ~3.0, the elastic pressure overcomes the mixing pressure. In the high swelling region, the elastic pressure dominates the order of the swelling ratios, which is the main reason for the abnormal order of *Q* among the Tetra-PAA-PEG gels.

**Figure 6:**
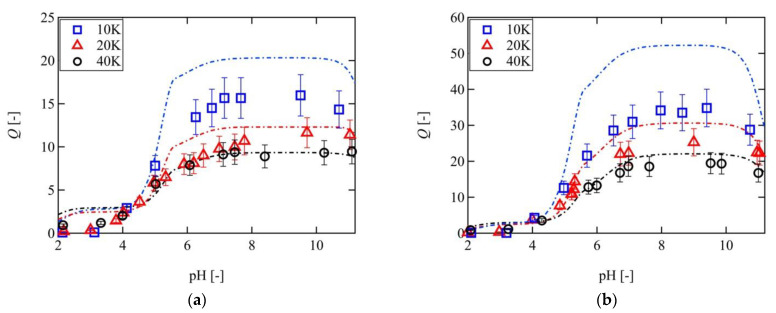
Comparison of the experimental results with the predictions made by the FRGDM model (dashed lines) at ionic strengths of (**a**) 10 mM, and (**b**) 2 mM for the 10 K (blue), 20 K (red), and 40 K (black) Tetra-PAA-PEG gels.

Figure 6 compares the experimental results of the 10 K, 20 K, and 40 K Tetra-PAA-PEG gels with the predictions made using the FRGDM model under ionic strengths of 2 and 10 mM. As indicated, the FRGDM model predictions reproduce well the swelling behavior of the 40 K Tetra-PAA-PEG gel. However, as *Q* becomes larger, the downward deviation from the theoretical lines becomes more pronounced, thereby indicating that the original FRGDM model fails to predict *Q* in the higher swelling regime (*Q* > 10).

### 3.4. Effect of the Finite Extensibility of the Polymer Chains on the Swelling Behavior

The overestimation of swelling can be attributed to a failure in describing the elastic pressure. The Neo–Hookean model considers the elastic free energy of ideal polymer networks with infinite extensibility [6,35,49]. Notably, the *Q* value of the 10 K Tetra-PAA-PEG gel in a salt solution with an ionic strength of 2 mM was significantly larger than that of the same gel under different external conditions, with the maximum value of *Q* reaching up to 35 times greater than that under the original conditions. Under such a large deformation, the distribution function of the polymer segments does not follow the Gaussian statistics, and the Neo–Hookean model is no longer applicable for describing the elastic free energy.

To include the effect of the finite extensibility of the network strands, we adopted the Gent model, which describes the effect of the finite extensibility by the minimum addition to the Neo–Hookean model [50]. The Gent model expresses the elastic free energy $\Delta F_{\mathrm{el}}$ as:

(15) $$\Delta F_{\mathrm{el}}=-\frac{V_{0}G_{0}}{2}\left(I_{m}-3\right)\ln\left(1-\frac{I_{1}-3}{I_{m}-3}\right)$$

where $V_{0}$ is the volume in the as-prepared state. $I_{1}$ is the first invariant of Green’s deformation tensor, and $I_{m}$ is the maximum value of $I_{1}$, where the stress becomes infinite. $I_{1}$ is expressed as [51]:

(16) $$I_{1}=\alpha_{x}{}^{2}+\alpha_{y}{}^{2}+\alpha_{z}{}^{2}$$

where $\alpha_{i}$ is the elongation ratio in the *i* axis (i = *x*, *y*, *z*) of the gel from the as-prepared state to the swollen state. By assuming isotropic deformation, the elongation ratios are related to *Q* as follows:

(17) $$\alpha_{x}=\alpha_{y}=\alpha_{z}=\alpha =Q^{\frac{1}{3}}={\left(\frac{V}{V_{0}}\right)}^{\frac{1}{3}}$$

where *V* is the volume of the gel in the swollen state. To estimate the value of $I_{m}$, we utilize the uniaxial ultimate elongation ratio $\alpha_{\max}$, which can be estimated from the network strand length. The detailed calculation is provided in Supporting Information Section 3. The elastic pressure can be obtained by the derivation of $\Delta F_{\mathrm{el}}$ from the Gent model (see Supporting Information Section 4 for details) and is given by:

(18) $$\Pi_{\mathrm{el}}=-\frac{I_{m}-3}{I_{m}-I_{1}}G_{0}Q^{-\frac{1}{3}}$$

**Figure 7:**
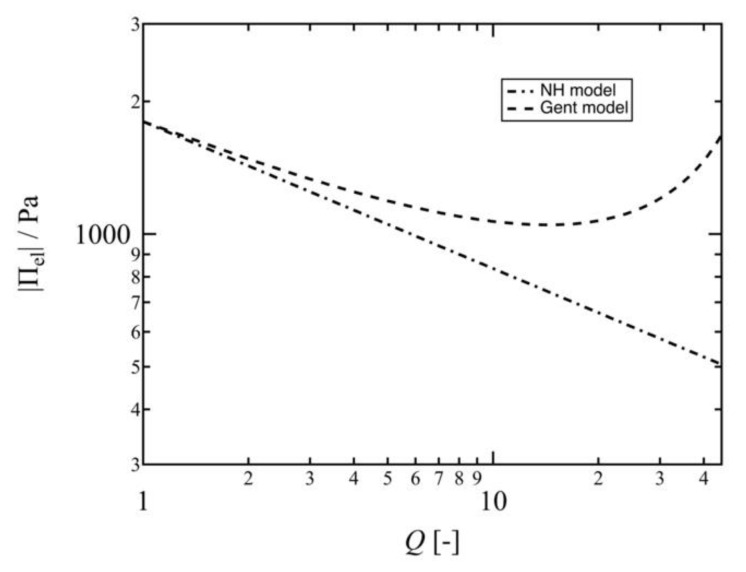
Swelling ratio (*Q*) dependence of the elastic pressure (|$\Pi_{\mathrm{el}}$|) of the 10 K Tetra-PAA-PEG gel. The dashed-dotted-dashed line represents |$\Pi_{\mathrm{el}}$| predicted by the Neo–Hookean model, while the dashed line represents |$\Pi_{\mathrm{el}}$ | predicted by the Gent model.

It is worth noting that $I_{m}$ is invariable, as calculated above, while $I_{1}$ is a function of *Q*. Thus, $\Pi_{\mathrm{el}}$ is a function of both Q and $G_{0}$. Figure 7 shows the swelling ratio dependence of the elastic pressure of the 10 K Tetra-PAA-PEG gel predicted by the Neo–Hookean and Gent models. The Gent model prediction is larger than the Neo–Hookean model prediction, and the gap between the |$\Pi_{\mathrm{el}}$| values of the two models become significant with an increase in *Q*. In the higher *Q* region (*Q* > 20), the Gent model predicts an upturn of |$\Pi_{\mathrm{el}}$| with swelling. Indeed, a similar upturn in *G* with swelling was experimentally observed in our previous study, and thus is not an unrealistic estimation [52].

**Figure 8:**
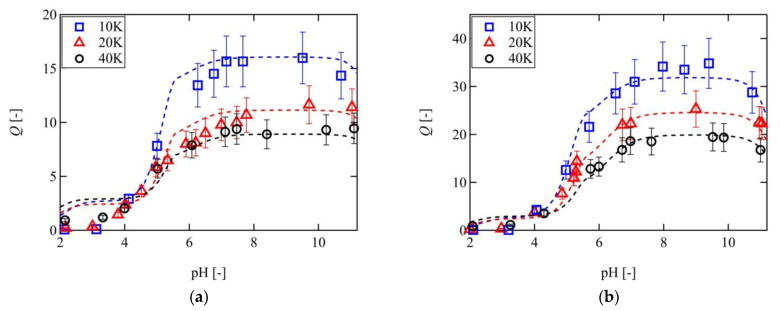
Comparison of the experimental results with the predictions made by the FRGDM model, adopting the Gent model to describe the elastic free energy (dashed lines) for the 10 K (blue), 20 K (blue), and 40 K (black) Tetra-PAA-PEG gels at ionic strengths of (**a**) 10 mM and (**b**) 2 mM.

Figure 8 compares the experimental results with the predictions made by the FRGDM model wherein the Gent model was adopted to describe the elastic free energy. As indicated, the predictions clearly reproduce the swelling behaviors of the various Tetra-PAA-PEG gels under all external conditions examined, including the condition where the original FRGDM model fails. It is worth noting that the downward deviation at low-pH regions was attributed to the formation of the aggregation structure through hydrogen bonds between PAA and PEG, which was reported elsewhere as well [25]. This good correspondence indicates that the finite extensibility effect is essential for describing the elastic free energy of a highly swollen gel.

## 4. Conclusions

We successfully fabricated a series of polyelectrolyte gels (Tetra-PAA-PEG gels) possessing alternating neutral/highly charged sequences by the combination of tetrathiol-terminated poly (ethylene glycol) (Tetra-PEG-SH) units and tetramaleimide-terminated poly (acrylic acid) (Tetra-PAA-MA) units. These gels exhibited tuned elastic moduli with a fixed crosslinking density and a fixed charged concentration. Subsequently, we investigated the swelling behaviors of the Tetra-PAA-PEG gels under various external conditions (pH: 2~11, ionic strength: 2 and 10 mM). It was found that the swelling ratios increased from 0.1 to 34.2 with increasing pH and decreasing ionic strength due to ionization of the PAA group. The prediction of the FRGDM model (i.e., a combination of the Flory–Rehner model with the Gibbs–Donnan effect and Manning’s counterion condensation model) deviated upward from the swelling of the Tetra-PAA-PEG gels in the high swelling region. Upon considering the finite extensibility effect in the predictions, the experimental results were well reproduced. These findings indicate the importance of finite extensibility for predicting the swelling properties of polyelectrolyte gels, and confirm that the modified FRGDM model is a promising model for this purpose. Investigation of the modified FRGDM model’s applicability for the popular gels with the uncontrolled fixed ions distribution is a future plan.

## 5. Materials and Methods

### 5.1. Fabrication of the Tetra-PAA-PEG Gels

Tetrathiol-terminated poly (ethylene glycol) (tetra-PEG-SH) was purchased from NOF Corporation (Tokyo, Japan). Tetramaleimide-terminated poly (acrylic acid) (Tetra-PAA-MA) was prepared from Tetraazide-terminated poly (*tert*-butyl acrylate) (Tetra-PtBuA-N_3_). Details regarding preparation of tetra-PAA and tetra-PEG precursors have been reported previously [24,53,54,55]. Equivalent moles of Tetra-PAA-MA and Tetra-PEG-SH were dissolved in a citrate-phosphate buffer solution with a pH and ionic strength of 5.9 and 100mM, respectively. The Tetra-PAA-PEG gels fabricated using Tetra-PEG-SH with *M*_w_ values of 10, 20, and 40 kg mol^−1^ are referred to as 10 K Tetra-PAA-PEG gel, 20 K Tetra-PAA-PEG gel, and 40 K Tetra-PAA-PEG gel, respectively. The *M*_w_ of the Tetra-PAA-MA component used for the preparation of all Tetra-PAA-PEG gels was 19 kg mol^−1^. To obtain the consistent initial fixed ion concentration, the same prepolymer molar concentrations were employed, and the strand lengths of the PEG were varied. In addition, the concentration of Tetra-PAA-MA was fixed at 60.0 g L^−1^ for all types of Tetra-PAA-PEG gel, while the concentrations of tetra-PEG-SH were set to 32.3, 64.6, and 129.3 g L^−1^ for the 10 K, 20 K, and 40 K Tetra-PAA-PEG gels, respectively. Equal amounts of the prepolymer solutions were mixed for 30 s and poured into a silicone mold at 25 °C. A minimum of 48 h reaction time was permitted for each gel to form.

### 5.2. Rheological Measurements after Gelation

The gel samples were prepared as disk films and set at the measuring plate of a rheometer (MCR301; Anton Paar, Graz, Austria) equipped with a parallel plate fixture with a diameter of 25 mm. The diameter and thickness of each sample in the as-prepared state were 40.0 mm and 1.0 mm, respectively. The angular frequency (ω) dependences of the storage modulus (*G*) and the loss modulus (*G*′) were measured with a strain amplitude (*γ*) of 1.0% at 25 °C. The oscillatory shear strain amplitudes were found to be within the range of the linear viscoelasticity for all tests.

### 5.3. Swelling Experiments

The gel samples employed for the swelling ratio measurements were prepared as rectangular films (length: 5.0 mm, thickness: 2.0 mm, width: 3.0 mm). The pH of the external solution was varied between 2.0 and 11.0. Each specimen was immersed in the desired solution for 48 h at 25 °C to reach the equilibrium swelling state. During this time, the pH of the external solution changed from its initial value due to the presence of carboxyl groups on the Tetra-PAA-MA polymer; thus, we titrated an 0.01 mol L^−1^ HCl (pH = 2.0) or 0.01 mol L^−1^ NaOH (pH = 12.0) solution into the external solution to adjust the pH back to its original value. In the ionic strength dependence measurements, the ionic strengths of the external solutions were varied from 10 to 1000 mM by the addition of different amounts of NaCl. The effects of the Na^+^ and Cl^−^ ions introduced by pH adjustment on the ionic strength of the external solutions can be neglected compared with the ionic strength tuning by NaCl itself.

The swelling ratios of the Tetra-PAA-PEG gels were investigated by measuring the volume changes of the samples using encoded stereomicroscopes (M165 C, Leica Co., Wetzlar, Germany). The swelling ratio (*Q*) was defined using the initial volume of the gel sample $V_{0}$ and the swollen volume of the gel sample $V_{s}$: *Q* $=\;V_{s}/V_{0}$. Generally, the swelling of the gels is isotropic. The volume change was estimated by using the change in the initial side length of the gel sample in the as-prepared state $L_{0}$ and the side length of the gel sample in the equilibrium swelling state $L_{s}$. Thus, the swelling ratio *Q* was expressed as *Q* $=\;\;{\left(L_{s}/L_{0}\right)}^{3}$. $L_{0}$ was 5.0 mm, while $L_{s}$ ranged from 2.2 to 17.3 mm depending on the external solution conditions.

## Supplementary Materials

The following are available online at https://www.mdpi.com/2310-2861/7/1/25/s1, Overlapping polymer volume fraction of the blend system of Tetra-PAA and Tetra-PAA; Relationship between the shear modulus in the as-prepared state and the concentration; Calculation of *I*_m_; and Calculation of Π_el_ of the Gent model.
