# Supplementary material for: Effect of Nonlinear Elasticity on the Swelling Behaviors of Highly Swollen Polyelectrolyte Gels
Source: Gels. 2021 Mar 1;7(1):25. doi: 10.3390/gels7010025 (PMC8005930; doi:10.3390/gels7010025)
Supplement: Supplementary file 1 [file gels-07-00025-s001.pdf]

Supporting Information

Effect of Nonlinear Elasticity on the Swelling Behaviors of Highly Swollen Polyelectrolyte Gels

Jian Tang[1], Takuya Katashima[1], Xiang Li[2], Yoshiro Mitsukami[3], Yuki Yokoyama[3], Ung-il Chung[1], Mitsuhiro Shibayama[2], and Takamasa Sakai[1*]*

Contents

1. **Overlapping polymer volume fraction of the blend system of Tetra-PAA and Tetra-PAA**
2. **Relationship between the shear modulus in the as-prepared state and the concentration**
3. **Calculation of I_m**
4. **Calculation of Π_{el} of the Gent model**

1. Overlapping polymer volume fraction of the blend system of Tetra-PAA and Tetra-PEG

The required quantities of Tetra-PAA-MA were dissolved in a citrate-phosphate buffer solution to obtain a polymer solution with concentrations ranging from 0 to 120 g L^{-1}. The pH and ionic strength of the buffer solution were 5.9 and 100 mM, respectively. The effect of acrylic acid ionization was suppressed by applying the buffer solution as a solvent. The viscosity η of the polymer solution was measured using a rheometer (MCR301; Anton Paar, Graz, Austria) with a cone plate at a constant shear rate of 100 s^{-1}, where the effect of the shear rate was confirmed to be negligible prior to the measurement. All experiments were performed at 25 ℃. The specific viscosity, η_{sp}, was expressed as:

$$\eta_{\mathrm{sp}} = \frac{\eta - \eta_0}{\eta_0} \tag{S-1}$$

where η_0 is the viscosity of the buffer solution.

Supporting Figure 1 shows the specific viscosities η_{sp} of the Tetra-PAA-MA polymer solutions as a function of the polymer concentration. From the crossover of the two fitting lines, the C^{*}_{PAA} of the Tetra-PAA-MA polymer was estimated to be 60 g L^{-1}, and the overlapping polymer volume fraction ϕ^{*}_{PAA} was calculated to be 0.050. The Tetra-PAA-PEG gel was fabricated from Tetra-PAA-MA and Tetra-PEG-SH prepolymers. Thus, the blend overlapping polymer volume fraction $\phi^{*}_{\mathrm{blend}}$ of the Tetra-PAA-PEG gel was determined using the ϕ^{*} and ϕ values of the Tetra-PAA-MA and Tetra-PEG-SH prepolymers as follows:

$$\phi^{*}_{\mathrm{blend}} = f_{\mathrm{PEG}}\phi^{*}_{\mathrm{PEG}} + f_{\mathrm{PAA}}\phi^{*}_{\mathrm{PAA}} \tag{S-2}$$

where ϕ^*_{PEG} is the overlapping polymer volume fraction of the Tetra-PEG prepolymer, which was measured in our previous research; [1] f_{PEG} and f_{PAA} are the volume fractions of Tetra-PEG-SH and Tetra-PAA-MA to the total polymer volume, respectively.

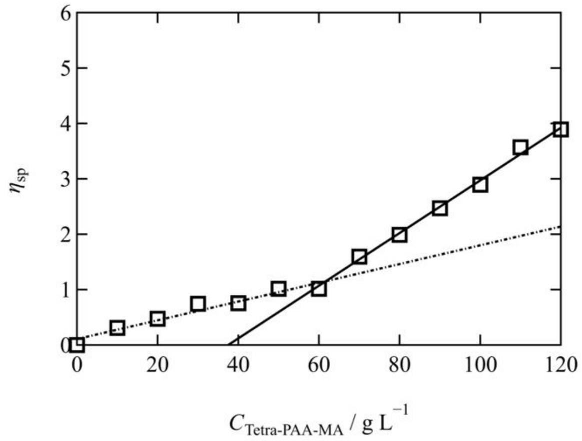

Supporting Figure 1. Specific viscosities of the Tetra-PAA-MA polymer solutions as a function of the polymer concentration. The overlapping concentration C^* was estimated to be the point at the intersection of the fitting lines of the lower and higher concentrations.

Supporting Table 1 shows the total polymer volume fraction ϕ_{total} and the overlapping polymer volume fraction ϕ^*_{blend} of the 10K, 20K, and 40K Tetra-PAA-PEG gels, respectively. The ϕ_{total} of the 10K Tetra-PAA-PEG gel was smaller than that of ϕ^*_{blend}, while the ϕ_{total} of the 20K and 40K Tetra-PAA-PEG gel was slightly larger than ϕ^*_{blend}.

Supporting Table 1. Polymer volume fractions (ϕ_{PEG}) of Tetra-PEG-SH, polymer volume fraction (ϕ_{PAA}) of Tetra-PAA-MA, total polymer volume fraction (ϕ_{total}) of the prepolymers inside the Tetra-PAA-PEG gels, and overlapping polymer volume fraction ($\phi^{*}_{\mathrm{blend}}$) of the 10K, 20K, and 40K tetra-PAA-PEG gels

	ϕ_{PEG} [-]	ϕ_{PAA} [-]	ϕ_{total} [-]	$\phi^{*}_{\mathrm{blend}}$ [-]
10K Tetra-PAA-PEG gel	0.014	0.025	0.039	0.054
20K Tetra-PAA-PEG gel	0.027	0.025	0.052	0.042
40K Tetra-PAA-PEG gel	0.053	0.024	0.077	0.025

2. Relationship between the shear modulus in the as-prepared state and the concentration

According to our previous studies [2], the G_0 value of the Tetra-PEG gel did not obey the simple rubber elasticity theories [1] due to the negative energetic contribution [3]. G_0 is given as:

$$G_0 = g(\nu - \mu) \tag{S-3}$$

where ν is the number per prepolymer of the elastically effective network strands, μ is the number per prepolymer of the active crosslink, and g is a pre-factor related to the energetic contribution. Our previous studies suggested that g is a function of ϕ/ϕ^{*}, as shown in Supporting Figure 2.

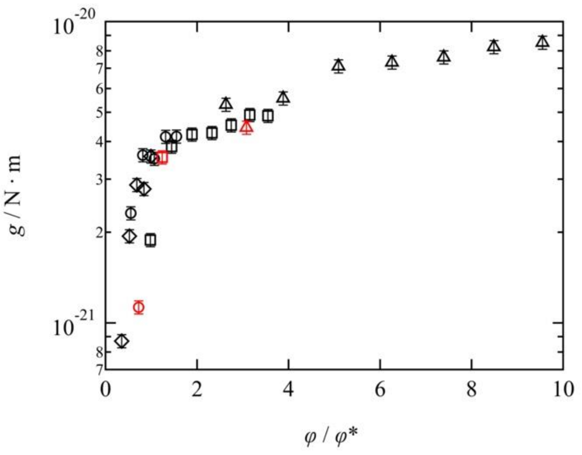

Supporting Figure 2. The value of g as a function of ϕ/ϕ^* for the Tetra-PEG gels (black: 5K, rhombus; 10K, circle; 20K, square; and 40K, triangle) and the Tetra-PAA-PEG gels (red: 10K, circle; 20K, square; and 40K, triangle). Data for the Tetra-PEG gels are replotted from Akagi et al. [2] with permission.

Supporting Figure 3 shows the reaction conversions of the 10K, 20K, and 40K Tetra-PAA-PEG gels, which ranged from 0.81 to 0.90. Adopting the Bethe approximation, ν and μ can be calculated as a function of the reaction conversion (p) [4,5]. Under stoichiometric mixing conditions, the AB-type reaction can be replaced by presuming that the reaction is an AA-type reaction of the 4-armed prepolymer. Thus, the probability of one arm of a 4-armed prepolymer not being connected to the percolated network, $P(F)$, is expressed as:

$$P(F) = \left(\frac{1}{p} - \frac{3}{4}\right)^{\frac{1}{2}} - \frac{1}{2} \tag{S-4}$$

Using equation (S-4), the probability of a 3-functional or 4-functional crosslink (i.e., $P(X_3)$ or $(P(X_4))$ can be derived from a 4-armed prepolymer as:

$$P(X_3) = \binom{4}{3} \cdot P(F) \cdot [1 - P(F)]^3 \tag{S-5}$$

$$P(X_4) = [1 - P(F)]^4 \tag{S-6}$$

Thus, μ and ν can be expressed as:

$$\mu = P(X_3) + P(X_4) \tag{S-7}$$

$$\nu = \frac{3}{2} \cdot P(X_3) + 2 \cdot P(X_4) \tag{S-8}$$

g for the Tetra-PAA-PEG gel was obtained by inserting equations (S-7) and (S-8) into equation (S-3).

The obtained values of g are plotted in Supporting Figure 2, which obeys the master curve of the Tetra-PEG gels. These agreements support that observation that the reduction of G_0 for the Tetra-PAA-PEG gels with shorter network lengths can be explained by the distance from ϕ^*.

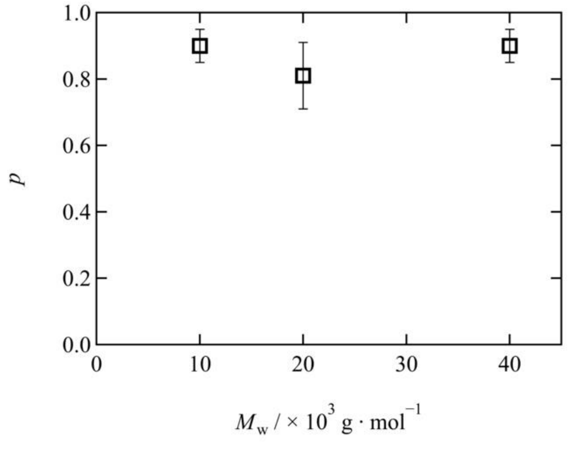

Supporting Figure 3. Reaction conversions (p) for the 10K, 20K, and 40K Tetra-PAA-PEG gels.

3. Calculation of I_m

According to the classical concept of elastomers [6], I_m is the value of I_1 at the ultimate deformation at which the network strands are fully stretched. Considering the

case in which the network strands are fully stretched, the uniaxial deformation ratio α_{max} is given as:

$$\alpha_{\mathrm{max}} = \frac{aN_{\mathrm{e}}}{aN_{\mathrm{e}}^{1/2}} = N_{\mathrm{e}}^{1/2} \qquad \text{(S-9)}$$

where a is the Kuhn length and N_{e} is the segment number of the network strand between the neighboring elastically effective junctions. Under this condition, the invariant is expressed as follows:

$$I_{\mathrm{m}} = \alpha_{\mathrm{max}}^{2} + 2\alpha_{\mathrm{max}}^{-1} \qquad \text{(S-10)}$$

It should be noted that equation (S-10) assumes uniaxial deformation, while the value of I_{m} does not depend on the deformation type.

In this study, the N_{e} value of the Tetra-PAA-PEG gel was calculated as the sum of the PAA and PEG units:

$$N_{\mathrm{e}} = \frac{M_{\mathrm{PAA}} b_{\mathrm{PAA}}}{4 m_{\mathrm{PAA}} a_{\mathrm{PAA}}} + \frac{M_{\mathrm{PEG}} b_{\mathrm{PEG}}}{4 m_{\mathrm{PEG}} a_{\mathrm{PEG}}} \qquad \text{(S-11)}$$

where M_{PAA} is the molecular weight of the Tetra-PAA-MA prepolymer, M_{PEG} is the molecular weight of the Tetra-PEG-SH prepolymer, m_{PAA} is the molecular weight of a monomeric PAA unit, m_{PEG} is the molecular weight of a monomeric PEG unit, and a_{PAA} and a_{PEG} are the Kuhn segment sizes of PAA and PEG, respectively. Substituting equations (S-9) and (S-11) into equation (S-10), I_{m} can be estimated. Supporting Table 2 summarizes the calculated results of N_{e}, α_{max}, and I_{m}.

Supporting Table 2. The molecular masses of Tetra-PEG-SH (M_{PEG}), of Tetra-PAA-MA (M_{PAA}), the degree of polymerization between the neighboring elastically effective junctions of the polymer network (N_{e}) of prepolymers inside the Tetra-PAA-PEG gels, the ultimate elongation ratio (α_{max}), and the maximum value of I_1 under the ultimate elongation ratio (I_{m}) of the 10K, 20K, and 40K Tetra-PAA-PEG gels

Sample	M_{PEG} [g/mol]	M_{PAA} [g/mol]	N_{e} [-]	$\alpha_{\max}$ [-]	I_{m} [-]
10K Tetra-PAA-PEG gel	1.0×10^4	1.9×10^4	53	7.3	53
20K Tetra-PAA-PEG gel	2.0×10^4	1.9×10^4	81	9.0	81
40K Tetra-PAA-PEG gel	4.0×10^4	1.9×10^4	1.4×10^2	12	1.4×10^2

4. Calculation of Π_{el} of the Gent model

Π_{el} is defined as a change in the elastic free energy (ΔF_{el}) when changing the number of solvent molecules (n_{B}) with a constant number of polymers in the system (n_{A}) and is expressed as:

$$\Pi_{\text{el}} = -\frac{1}{V_1}\left(\frac{\partial \Delta F_{\text{el}}}{\partial n_{\text{B}}}\right)_{n_{\text{A}}} \tag{S-12}$$

where N_{A} is Avogadro's constant, and V_1 is the molar volume of the solvent. Thus, N_{A}/V_1 is the number of solvent molecules per unit volume. According to the Gent model, the elastic free energy ΔF_{el} is expressed as:

$$\Delta F_{\text{el}} = -\frac{V_0 G_0}{2}(I_{\text{m}} - 3)\ln\left(1 - \frac{I_1 - 3}{I_{\text{m}} - 3}\right) \tag{S-13}$$

where G_0 and V_0 are the shear modulus and volume of the gel in the as-prepared state, respectively. In addition, I_1 is the first invariant of Green's deformation tensor, I_{m} is the maximum value of I_1 where the stress becomes infinite, and I_1 is expressed as:

$$I_1 = {\alpha_x}^2 + {\alpha_y}^2 + {\alpha_z}^2 \tag{S-14}$$

where α_i is the elongation ratio in the i axis (i=x, y, z) of the gel in the swollen state.

The elongation ratios are obtained by assuming isotropic deformation, which is given as:

$$\alpha_x = \alpha_y = \alpha_z = \alpha = Q^{\frac{1}{3}} = \left(\frac{V}{V_0}\right)^{\frac{1}{3}} \tag{S-15}$$

where Q and V are the swelling ratio and volume in the swollen state, respectively. Substituting equations (S-13), (S-14), and (S-15) into equation (S-12), the following equation is obtained:

$$\Pi_{\mathrm{el}} = -\frac{1}{V_1}\left(\frac{\partial \Delta F_{\mathrm{el}}}{\partial n_{\mathrm{B}}}\right)_{n_{\mathrm{A}}} = -\frac{1}{V_1}\left(\frac{\partial \Delta F_{\mathrm{el}}}{\partial \alpha}\right)_{n_{\mathrm{A}}}\left(\frac{\partial \alpha}{\partial n_{\mathrm{B}}}\right)_{n_{\mathrm{A}}} = -\frac{3\alpha V_0 G_0}{V_1}\frac{I_{\mathrm{m}} - 3}{I_{\mathrm{m}} - I_1}\left(\frac{\partial \alpha}{\partial n_{\mathrm{B}}}\right)_{n_{\mathrm{A}}} \tag{S-16}$$

Here,

$$\alpha^3 = \frac{V}{V_0} = \frac{V_0 + n_{\mathrm{B}} V_1}{V_0} \tag{S-17}$$

Therefore, Π_{el} can be rewritten as

$$\Pi_{\mathrm{el}} = -\frac{I_{\mathrm{m}} - 3}{I_{\mathrm{m}} - I_1} G_0 Q^{-\frac{1}{3}} \tag{S-18}$$

In equation (S-18), the term $G_0 Q^{-\frac{1}{3}}$ is equal to the shear modulus in the swollen state. Thus, Π_{el} is experimentally validated by measuring the shear modulus.

References

1. Akagi, Y.; Gong, J.P.; Chung, U.Il.; Sakai, T. Transition Between Phantom and Affine Network Model Observed in Polymer Gels With Controlled Network Structure. *Macromolecules* **2013**, 46 (3), 1035–1040.

2. Yoshikawa, Y.; Sakumichi, N.; Chung, U.Il.; Sakai, T. Connectivity Dependence of Gelation and Elasticity in AB-Type Polymerization: An Experimental Comparison of the Dynamic Process and Stoichiometrically Imbalanced Mixing. *Soft Matter* **2019**, *15* (25), 5017–5025.

3. Yoshikawa, Y.; Sakumichi, N.; Chung, U.; Sakai, T. Negative Energy Elasticity in a Rubber-Like Gel. *Phys. Rev. X* **2021**.

4. Macosko, C.W.; Miller, D.R. A New Derivation of Average Molecular Weights of Nonlinear Polymers. *Macromolecules* **1976**, *9* (2), 199–206.

5. Miller, D.R.; Macosko, C.W. A New Derivation of Post Gel Properties of Network Polymers. *Macromolecules* **1976**, *9* (2), 206–211.

6. Obukhov, S.P.; Rubinstein, M.; Colby, R.H. Network Modulus and Superelasticity. *Macromolecules* **1994**, 27 (12), 3191–3198.
